# Supplementary figures and images for: High prevalence of Strongyloides stercoralis in people living with HIV: A critical health challenge in the Peruvian Amazon Basin
Source: PLoS Negl Trop Dis. 2025 Jul 15;19(7):e0013231. doi: 10.1371/journal.pntd.0013231 (PMC12262856; doi:10.1371/journal.pntd.0013231)

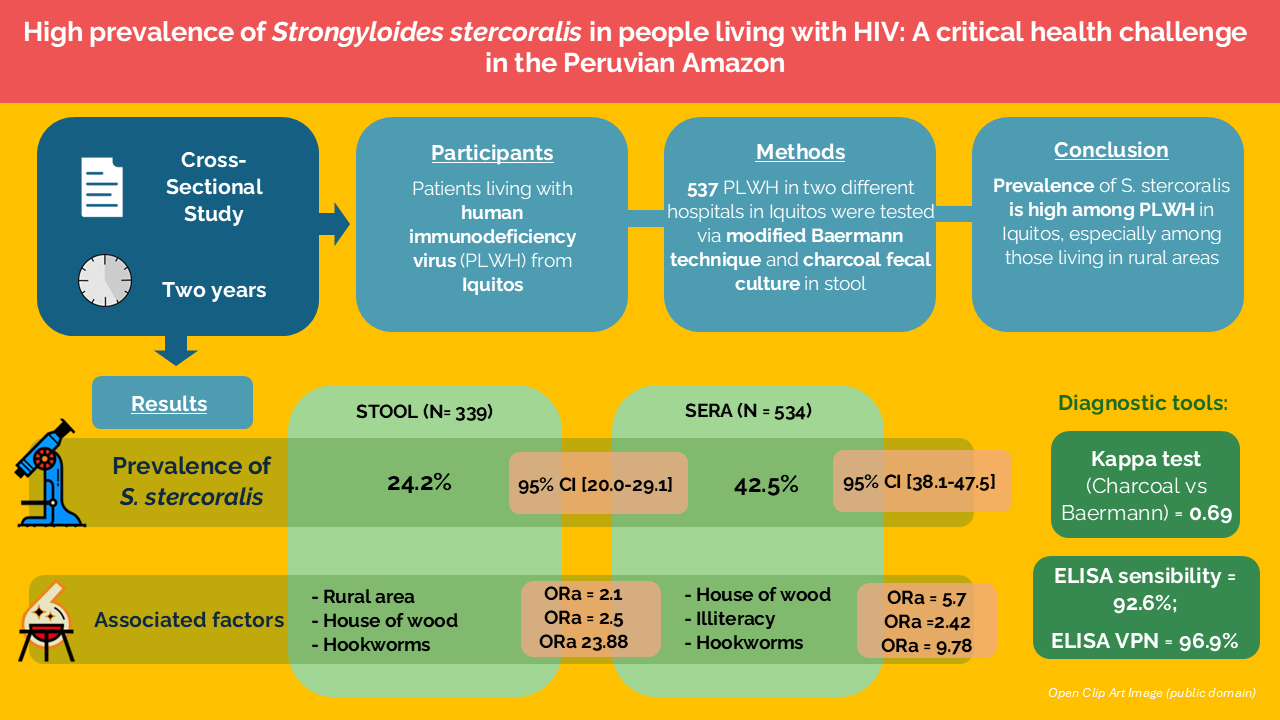

Supplement: S1 File — (TIF) [file pntd.0013231.s001.tif]
