# Supplementary material for: High prevalence of Strongyloides stercoralis in people living with HIV: A critical health challenge in the Peruvian Amazon Basin
Source: PLoS Negl Trop Dis. 2025 Jul 15;19(7):e0013231. doi: 10.1371/journal.pntd.0013231 (PMC12262856; doi:10.1371/journal.pntd.0013231)
Supplement: Attachment — Submitted filename: Reviewer report.docx [file pntd.0013231.s002.docx]

Dear authors, this paper addresses an important issue, not only because of the coinfection HIV- S stercoralis, but also because it provides data about strongyloidiasis prevalence. However, I think the paper can be improved if you accept the next comments, if worthy and appropriate for you.

- Line 85 (abstract): “…as the parasitological gold standard…” As there is no gold standard diagnosis for S. stercoralis, I will advise to change by “more sensitive” parasitological method , to avoid confusion of concepts
- Line 117: “*Strongyloidiasis is clinically important because*…” I think the expression *clinically* is not correct, as strongyloidiasis is pauci-symptomatic. Maybe, epidemiologically, or just to say important.
- Line 133-134: “To the best of our knowledge, no published parasitological or Sero epidemiological studies exist on S. stercoralis infection in PLWH in Peru.”

See García C, Rodríguez E, Do N. López de Castilla D, Terashima a, Gotuzzo E. intestinal parasitosis in patients with HIV-AIDS. Rev Gastroenterol Peru. 2006;26(1):21–24

- Line 139: “…is not clinically available…” better to say …is not available
- Lines 142-143: “Overall, no available diagnostic tests can serve as a *gold standard* for the diagnosis of strongyloidiasis” I would advise to precise that a combination of techniques is the approach which allows a higher sensitivity (see Diagnostic methods for the control of strongyloidiasis, Virtual meeting, 29 September 2020. Geneva: World Health Organization; 2020.)
- Line 150: “…prospective, cross-sectional study …” The study design cannot be both prospective and cross-sectional!
- Line 183: “…to observe adult larvae before…” Do you mean filariform larvae?? Or adult???
- Line 193: “Considering the anticipated high seroprevalence and…” as you are including outpatients and inpatients and the last are the most representative in the sample, it is difficult to understand why you anticipated high seroprevalence ¿?
- Lines 279-280: “ WHO (…) emphasize the need for clear guidelines on whether preventive treatment for strongyloidiasis should be recommended,…” Actually, the reference you include (15) is the WHO guidelines, even though the guideline accepts the need of more evidence for strong recommendation

Major comments

- In the discussion, ELISA’s specificity and negative predictive values are assumed as high in the sample. However, severely immunosuppressed patients will have impaired their immunological response. The fact that serology can be inconclusive has been highlighted in these patients. (Nabha L, Krishnan S, Ramanathan R, et al. Prevalence of Strongyloides stercoralis in an urban US AIDS cohort. *Pathog Glob Health*. 2012;106(4):238-244. doi:10.1179/2047773212Y.0000000031). In the discussion you mention (line 359) that “…In our study, AIDS stage was not significantly associated with S. stercoralis stool positivity or seropositivity. However, the **Current viral load > 2000** was significantly associated with S. stercoralis serological status (table 4). I think it is important to include and discuss this issue. In this line, I would advise you to reconsider also the sentence in the abstract, about the PNV, Line 98, “…..which is useful for ruling out the presence of active infection” ¿?.

Also, direct observation was used in the analysis of the samples, but there is no mention of other STH or parasites. However, in view of the incidence of diarrhoea in the sample (table 1) and considering that the S. stercoralis infection is pauci-symptomatic, it would be important to mention that other STH, e.g., hookworm, can be co-infecting the patients, and the relationship with the positive serology in those patients, that is, the impact of this fact in the sensitivity of the test.

- Lines 92-95 (abstract)Lines 254-255: “risk factors for stool positivity included living in a rural area (adjusted OR: 2.16), whereas significant risk factors for both stool and seropositivity included living in a house made of wood/leaves (adjusted ORs: 2.10 and 2.48, respectively) or in the Loreto Regional Hospital catchment area (adjusted ORs: 5.66 and 5.37, respectively). Is the Loreto Regional Hospital catchment area an urban area? Are houses in rural areas made of different materials than wood/leaves? I mean, are those risk factors logical or are they confusing factors? To explain more in detail in the discussion
- 368-371: “ treatment in the previous 6 months was a protective factor for *S. stercoralis* stool positivity in the bivariable analysis but not in the multivariate analysis [32], probably because ivermectin (the treatment of choice for strongyloidiasis) use was uncommonly reported by our study participants…” I do not understand what this means exactly. Maybe you can rephrase it. Also, Albendazole can be effective for strongyloidiasis, if administered twice in 3 days. Maybe you will have this information ¿?
- Line 345: “…have hypothesized … ” Hypothesized??
- Lines 374-375: “This dual methodology enhances diagnostic accuracy by addressing both

active infections and past or subclinical exposure” I think this sentence is not correct. First, it is well known that the strongyloidiasis diagnosis benefits from a combination of techniques. Second, you cannot differentiate between active infections and past or subclinical exposure in a population living conditions you have describe as poor, related to hygiene and sanitation, where re-infection will be common.

Lines 383-385: “….as PCR has a higher sensitivity than either of the parasitological methods we employed and could identify low-level infections” This is not correct; even though the PCR is slightly more sensitive that Baermann technique in most of the studies, the test is negative in a high proportion of Baermann positives (see. Epidemiology of intestinal helminthiases in a rural community of Ethiopia: Is it time to expand control programs to include Strongyloides stercoralis and the entire community? *PLoS neglected tropical diseases*, *14*(6), e0008315. https://doi.org/10.1371/journal.pntd.0008315)
