# Supplementary material for: High prevalence of Strongyloides stercoralis in people living with HIV: A critical health challenge in the Peruvian Amazon Basin
Source: PLoS Negl Trop Dis. 2025 Jul 15;19(7):e0013231. doi: 10.1371/journal.pntd.0013231 (PMC12262856; doi:10.1371/journal.pntd.0013231)
Supplement: Attachment — Submitted filename: Response to Reviewers.docx [file pntd.0013231.s004.docx]

**Response to Reviewers**

Esteemed editor and reviewers, we would like to thank your time and effort in considering and reviewing our manuscript, and the clarifications requested, which undoubtedly have enriched the quality of our work. We have thoroughly reviewed the article, adapting it to all the recommendations. Changes are reflected in the text in red.

Subsequently, we will answer and clarify individually each of the points requested:

**Reviewer 1**

1. **Methods: Is the study design appropriate to address the stated objectives? THE DESCRIPTION OF STUDY DESIGN SHOULD BE REVISED**

*Answer:* *Thanks for this point. Following your indications in the comments below, we have changed the description of the study, removing the word “prospective” to avoid inaccuracies.*

*(Line 173): “This was an observational ~~prospective,~~ cross-sectional study of PLWH receiving care at one of two hospitals in Iquitos,…”*

2. **Results**: Not comments about these field were done by Reviewer 1.
3. **Conclusions**: **Do the authors discuss how these data can be helpful to advance our understanding of the topic under study? I think the data should be discussed in more detail in some important issues**

*Answer: Thank you for this feedback. As it is explained below, we have modified the discussion with the suggestions provided.*

4. **Editorial data:** Not comments about these field were done by Reviewer 1.
5. **Summary and general comments:** **I think the paper can be improved if you accept the next comments, if worthy and appropriate for you:**

**• Line 85 (abstract): “…as the parasitological gold standard…” As there is no gold standard diagnosis for S. stercoralis, I will advise to change by “more sensitive” parasitological method , to avoid confusion of concepts.**

*Answer: We absolutely agree with this comment, so we had made the proposed change:*

*(Line 84-85) “…via the modified Baermann technique and/or charcoal fecal culture as ~~the more~~ highly sensitive parasitological techniques ~~gold standard~~.”*

• **Line 117: “Strongyloidiasis is clinically important because…” I think the expression clinically is not correct, as strongyloidiasis is pauci-symptomatic. Maybe, epidemiologically, or just to say important.**

*Answer: Thank you for the clarification. The word “clinically” has been removed:*

*(Line 130) “Strongyloidiasis is ~~clinically~~ important because…”*

• **Line 133-134: “To the best of our knowledge, no published parasitological or Sero epidemiological studies exist on S. stercoralis infection in PLWH in Peru.”
See García C, Rodríguez E, Do N. López de Castilla D, Terashima a, Gotuzzo E. intestinal parasitosis in patients with HIV-AIDS. Rev Gastroenterol Peru. 2006;26(1):21–24**

Answer: Thanks for allowing us to make this clarification. A mistake on our part was done here, since we wanted to refer to the fact that there were no studies carried out in the “Peruvian Amazon”, rather than in “Peru”. The mistake has been corrected.

*(Lines 155-156): “To the best of our knowledge, no published parasitological or sero-epidemiological studies exist on S. stercoralis infection in PLWH in the Peruvian Amazon ~~Peru~~.”*

•**Line 139: “…is not clinically available…” better to say …is not available**

*Answer: Thank you for your comment. The word “clinically” has been removed:*

*(Line 161) “… procedure is not ~~clinically~~ available in most patient care settings.”*

**• Lines 142-143: “Overall, no available diagnostic tests can serve as a gold standard for the diagnosis of strongyloidiasis” I would advise to precise that a combination of techniques is the approach which allows a higher sensitivity (see Diagnostic methods for the control of strongyloidiasis, Virtual meeting, 29 September 2020. Geneva: World Health Organization; 2020.)**

*Answer: Thank you for your comment. We have change the sentence to make it more accurate, as suggested.*

*(Lines 164-165): “Overall, only a combination of techniques can be accepted as highly sensitive ~~no available diagnostic tests can serve as a gold standard~~ for the…”*

**• Line 150: “…prospective, cross-sectional study …” The study design cannot be both prospective and cross-sectional!**

*Answer: We are very grateful to you for your comment. As you mentioned, since this is a cross-sectional study, it does not involve cohort follow-up. Our reference to "prospective" refered to the data collection process, as the patients were included and evaluated specifically for our study (no retrospective data was used). However, since this term is contradictory, it has been removed from the text.*

*(Line 173): “This was an observational ~~prospective,~~ cross-sectional study of PLWH receiving care at one of two hospitals in Iquitos,…”*

• **Line 183: “…to observe adult larvae before…” Do you mean filariform larvae?? Or adult???**

*Answer: Thank you for allowing us to make this clarification. Dancescu's culture conditions allow us to observe adult larvae, both free-living and filariform larvae (the infective form). However, rhabditiform larvae are difficult to see due to their smaller size. We have pointed this out in the text.*

*(Line 207): “…stercoralis adult larvae (free-living state or filariform larvae) on the second, fourth…”*

• **Line 193: “Considering the anticipated high seroprevalence and…” as you are including outpatients and inpatients and the last are the most representative in the sample, it is difficult to understand why you anticipated high seroprevalence ¿?**

*Answer: We truly apologize for this error on our part, since the most representative in the sample were outpatients, but there was an error in Table 1. It has been corrected below:*

*(Line 262):*

| Table 1. Epidemiological characteristics of study participants, divided by those with available serum specimens and those with stool specimens | | |
| --- | --- | --- |
| Variables | **Patients with serum specimen** (N = 534) | **Patients with stool specimen** (N = 339) |
| Male, % (n/N) | 66.1 (353/534) | 64.3 (218/339) |
| Age, median (IQR), years | 41 (32, 49) | 41 (32, 48) |
| Hospital attended, % (n/N) |  |  |
| Regional Hospital of Loreto | 77.9 (416/534) | 84.7 (287/339) |
| ~~Outpatient~~ Inpatient | 1.5 (8/534) | 0.3 (1/339) |
| ~~Inpatient~~ Outpatient | 76.4 (408/534) | 84.4 (286/339) |
| Hospital of Iquitos (~~Inpatient~~ Outpatient) | 22.1 (118/534) | 15.3 (52/339) |

• **Lines 279-280: “ WHO (…) emphasize the need for clear guidelines on whether preventive treatment for strongyloidiasis should be recommended,…” Actually, the reference you include (15) is the WHO guidelines, even though the guideline accepts the need of more evidence for strong recommendation**

*Answer: We agree with your comment. We wanted to highlight that the infection is in the spotlight of international organizations such as the WHO, and a target to improve inside the health goals for 2030. It is therefore important to expand knowledge about it due to its relevance in the field of public health. Given the confusion, we have rephrased the sentence. Because of the comments of another Reviewer, this lines have been changed to the Introduction section.*

*(Lines 136-140): “Specifically, the organization* *denounces the need for more evidence to optimize public health programs for strongyloidiasis [5]. ~~WHO 2030 targets~~ ~~emphasize the need for clear guidelines on whether preventive treatment for strongyloidiasis should be recommended, creating an opportunity to incorporate strongyloidiasis control into ongoing public health efforts for neglected tropical diseases.”~~*

• **In the discussion, ELISA’s specificity and negative predictive values are assumed as high in the sample. However, severely immunosuppressed patients will have impaired their immunological response. The fact that serology can be inconclusive has been highlighted in these patients. (Nabha L, Krishnan S, Ramanathan R, et al. Prevalence of Strongyloides stercoralis in an urban US AIDS cohort. Pathog Glob Health. 2012;106(4):238-244. doi:10.1179/2047773212Y.0000000031).**

*Answer: Thank you for your point. We have highlighted this important comment in the Discussion section.*

*(Lines 378-380): “On the other hand, sensitivity of ELISA could be affected in AIDS patients, who may have a diminished immune response to the parasite [40], even if this population in our cohort was low (13.9%).”*

**In the discussion you mention (line 359) that “…In our study, AIDS stage was not significantly associated with S. stercoralis stool positivity or seropositivity. However, the Current viral load > 2000 was significantly associated with S. stercoralis serological status (table 4). I think it is important to include and discuss this issue. In this line, I would advise you to reconsider also the sentence in the abstract, about the PNV, Line 98, “…..which is useful for ruling out the presence of active infection” ¿?.**

*Answer: Thank you for your comments. Even if viral load > 2000 was a significant risk factor in the bivariate analysis, it was not confirmed in the multivariate analysis. It may be because both, data about viral load and CD4, were not always available, and this could affect the significance. We have explained our main hypothesis in the text, in order to better understanding of this issue. About the high negative predictive value of the ELISA serology, we were of course explaining our personal results in this cohort, where, comparing with the stool results, NPV of serology resulted to be >96%. However, we have also modified the mentioned sentence in Line 98, as can be misleading.*

*(Line 417-428): …“while a viral load >2000 copies/ml was significatively associated with seropositivity in the bivariate, but not in the multivariate analysis. As a mere hypothesis, detectable viral load could be related to a recent diagnosis or lack of adherence to the antiretroviral treatment, what, confusingly, can lead to a greater intestinal parasite infection. As a big number of data were lost in CD4 and viral load variables, a higher sample need to be analyzed to draw stronger conclusions about the relation between an impaired immunovirological control in HIV and acquisition of the infection. ~~This could be because our patients attended their routine check-ups, and therefore had better control of the infection~~. ~~Prior studies have also been inconclusive regarding the interaction between HIV and S. stercoralis infections, keeping in mind that HIV does not specifically impair Th2 immunity, in contrast to the known detrimental impacts of HTLV-1 infection and corticosteroid treatment [17]. However, in our study~~, Other previously described risk factors, including a non-heterosexual sexual orientation [24,45], ~~viral load > 2000,~~ …”*

*(Line 454-455): “Furthermore, we lost a significant data about CD4 and viral load, important to draw conclusions about risk factors in PLWH with poor immunovirological control…”*

*(Line 98-103): “Conclusion: The prevalence of S. stercoralis is high among PLWH in Iquitos, especially among those living in rural areas. The negative predictive value of the S. stercoralis ELISA ~~could be an excellent negative predictive value~~ was high, although this result should be taken with caution in severe immunosuppression. ~~was a good tool for population-based screening because it has high negative predictive value., which is useful for ruling out the presence of active infection.”~~*

**Also, direct observation was used in the analysis of the samples, but there is no mention of other STH or parasites. However, in view of the incidence of diarrhoea in the sample (table 1) and considering that the S. stercoralis infection is pauci-symptomatic, it would be important to mention that other STH, e.g., hookworm, can be co-infecting the patients, and the relationship with the positive serology in those patients, that is, the impact of this fact in the sensitivity of the test.**

*Answer: As you conveniently mentioned in this paragraph, we have also registered other intestinal parasites present in the stool sample, even if we had not included the results here so as not to prolong the text. But, as it is an interesting data, we have already added our results and discussed them. In both, the bivariate and the multivariate analysis, a significative relationship between hookworms and Strongyloides stercoralis in both, stool infection and seropositivity has been found. Previous authors have discuss this in depth, explaining that the distribution of both species could frequently be overlapped, given their biological and epidemiological similarity, as both infections are acquired by contact of the larvae with the skin when walking barefoot or bathing in rivers (Krolewiecki A, Nutman TB. Strongyloidiasis. Infect Dis Clin North Am. marzo de 2019;33(1):135-51. Fleitas PE, Travacio M, Martí-Soler H, Socías ME, Lopez WR, Krolewiecki AJ ; The Strongyloides stercoralis-hookworms association as a path to the estimation of the global burden of strongyloidiasis: A systematic review. Cappello M, editor. PLoS Negl Trop Dis. 13 de abril de 2020;14(4):e0008184; Bern C, Chavez CB, Leung F, Sanchez GM, Oré CV, Gilman RH, et al. SEROEPIDEMIOLOGY OF STRONGYLOIDIASIS IN THE PERUVIAN AMAZON. Am J Trop Med Hyg. 1 de enero de 2006;74(1):97-102). Some of this studies have therefore proposed estimating S. stercoralis from the hookworm load at the study site, in order to overcome the obstacle of the diagnostic complexity of strongyloidiasis. A cross reaction with serology because of hooworms has been feared, but when we analyzed the sensibility of the test, it remained as high as 90%, with a negative predictive value of 95%, as only one hookworm infection was found to have a positive serology in absence of S. stercoralis stool infection. In fact, as co-infection with both microorganisms is frequent, S. stercoralis could be harder to find, and treatment with Ivermectine has no major side effects, some authors suggest to treat patients with hookworms also with Ivermectine. We have included all this explanation in the abstract, the results and the discussion:*

*(Line 92-97): “Significant risk factors for stool positivity included living in a rural (unpaved) area (adjusted OR: ~~2.16~~ 1.86), whereas significant risk factors for both stool and seropositivity included living in a poor house (made of wood/leaves) (adjusted ORs: ~~2.10~~ 2.18 and 2.48, respectively), ~~or~~ in the Loreto Regional Hospital catchment area (adjusted ORs: 5.66 and 5.37, respectively), or being infected by hookworms in stool (adjusted ORs: 23.88 and 9.78, respectively). Having a low level of studies was associated with seropositivity (adjusted OR 2.42)”.*

*(Line 98-100): “Conclusion: The prevalence of S. stercoralis is high among PLWH in Iquitos, especially among those living in conditions of socioeconomic vulnerability ~~rural areas~~ or co-infected with hookworms.”*

*(Line 242-243): “Same analysis was realized also including other helminths infections, in other to rule out cross-reactions”.*

*(Line 274-280):*

*3.3. Other Helminths isolated in stool examination*

*Among the 339 patients with stool specimen, 17 (5.0%) were positive for hookworms, 10 (2.9%) for Ascaris lumbricoides, 2 (0.6%) for Hymenolepis nana and 1 (0.3%) for Trichuris trichiura.*

*Among the 82 patients who were positive for S. stercoralis infection in stool, 15 (18.2%) were also co-infected by hookworms, 6 (7.3%) were coinfected by Ascaris lumbricoides, and 1 (1%) was co-infected by Trichuris trichiura.*

*(Lines 297-303): “…After adjusting by sex, age, and variables with p values ≤ 0.05 in the bivariate analysis (****Table 4****), ~~two~~ four variables had a significant association with S. stercoralis seropositivity: belonging to Loreto Regional Hospital catchment area (adjusted OR~~: 5.37~~ 3.88), ~~and~~ living in a house made of wood/leaves (adjusted OR: ~~2.48~~ 2.82), having hookworms in stools (adjusted OR: 9.78) ~~(~~****~~Figure 3b~~****~~),~~ and having a low level of education (illiteracy or primary school) (adjusted OR 2.42) (****Figure 3b****). The model’s Cox–Snell R^2^ value was ~~0.08~~ 0.17 and the Nagelkerle R^2^ value was ~~0.12~~ 0.23, with an AUC of ~~0.69~~ 0.72 (95% CI ~~0.62–0.76~~ 0.67 – 0.78, p < 0.001).*

*(Lines 314-317): Only one hookworm infection was found to have a positive serology in absence of S. stercoralis stool infection. A cross reaction with serology because of hookworms has been feared, but when we analyzed the sensitivity of the test including this variable, it remained as high as 90% (IC:0.81-0.95), with a negative predictive value of 95% (0.91-0.98).*

*(Lines 408-415): “Hookworm infection was significantly related to infection in stool or seropositivity by S. stercoralis. This have been discussed in previous studies, as the distribution of both species could frequently be overlapped, given their biological and epidemiological similarity, being suggested as a reliable path to the estimate the global burden of strongyloidiasis [10,44].” Although there was an understandable fear of cross-reactions in serology due to other helminths, only one case showed a positive serology with stool positivity for hookworm rather than S. stercoralis, with no impact on the ELISA sensitivity/negative predictive values.*

*** Note to the reviewer: When the multivariate analysis was repeated with the new parameters, a significant value was obtained for the variable "low educational level," which had not been found significant in the first analysis. This is a logical risk factor for an infection associated with low socioeconomic status, so we have added this finding to the text:

*(Lines 91-100):* “…*Significant risk factors for stool positivity included living in a rural (unpaved) area (adjusted OR: ~~2.16~~ 1.86), whereas significant risk factors for both stool and seropositivity included living in a poor house (made of wood/leaves) (adjusted ORs: ~~2.10~~ 2.18 and 2.48, respectively), ~~or~~ in the Loreto Regional Hospital catchment area (adjusted ORs: 5.66 and 5.37, respectively), or being infected by hookworms in stool (adjusted ORs: 23.88 and 9.78, respectively). Having a low level of studies was associated with seropositivity (adjusted OR 2.42).*

*Conclusion: The prevalence of S. stercoralis is high among PLWH in Iquitos, especially among those living in conditions of socioeconomic vulnerability ~~rural areas~~ or co-infected with hookworms.”*

*(Lines 286-293): “After adjusting for sex, age, and variables with p values ≤ 0.05 in the bivariate analysis* ***(Table 3),*** *the characteristics most strongly associated with parasitological infection of S. stercoralis in PLWH were belonging to the Loreto Regional Hospital catchment area (adjusted OR: ~~5.66~~ 5.43), living in a rural area (adjusted OR: ~~2.16~~ 1.86), ~~and~~ living in a house made of wood/leaves (adjusted OR: ~~2.10~~ 2.18) and having hookworms in stools (adjusted OR: 23.88) (****Figure 3a****). The model’s Cox–Snell R^2^ value was ~~0.11~~ 0.18 and the Nagelkerle R^2^ value was ~~0.17~~ 0.27, with an AUC of ~~0.73~~ 0.76 (95% CI ~~0.67–0.79~~ 0.70 – 0.82, p < 0.001).”*

*(Lines 297-303): “ After adjusting by sex, age, and variables with p values ≤ 0.05 in the bivariate analysis (****Table 4****), ~~two~~ four variables had a significant association with S. stercoralis seropositivity: belonging to Loreto Regional Hospital catchment area (adjusted OR~~: 5.37~~ 3.88), ~~and~~ living in a house made of wood/leaves (adjusted OR: ~~2.48~~ 2.82), having hookworms in stools (adjusted OR: 9.78) ~~(~~****~~Figure 3b~~****~~),~~ and having a low level of education (illiteracy or primary school) (adjusted OR 2.42) (****Figure 3b****). The model’s Cox–Snell R^2^ value was ~~0.08~~ 0.17 and the Nagelkerle R^2^ value was ~~0.12~~ 0.23, with an AUC of ~~0.69~~ 0.72 (95% CI ~~0.62–0.76~~ 0.67 – 0.78, p < 0.001).”*

*(Lines 395-400): “…stool positivity was higher among PLWH living in a house made of wood/leaves (considering this an indicator of low economic power), or in a rural area (considering here all the unpaved streets, regardless of the type of material of the house), or with a low level of education, which tallies with well-known risk factors such as poor sanitary conditions, frequent contact with fecal contaminated soil due to lifestyle habits, and poor healthcare access and, encompassing this, with a socioeconomic vulnerability.”*

*The Tables 3, 4 and the Figure 3a - 3b have been changed adding this data.*

**• Lines 92-95 (abstract) Lines 254-255: “risk factors for stool positivity included living in a rural area (adjusted OR: 2.16), whereas significant risk factors for both stool and seropositivity included living in a house made of wood/leaves (adjusted ORs: 2.10 and 2.48, respectively) or in the Loreto Regional Hospital catchment area (adjusted ORs: 5.66 and 5.37, respectively). Is the Loreto Regional Hospital catchment area an urban area? Are houses in rural areas made of different materials than wood/leaves? I mean, are those risk factors logical or are they confusing factors? To explain more in detail in the discussion**

*Answer: Again, thank you for allowing us to make this clarification. A rural area was considered if ground was unpaved, due to the higher risk of S. stercoralis acquisition. This areas may or may not have wood houses, depending on the family possibilities, so this materials talk about the economic power of the people living there. Finally, Loreto Regional Hospital is an urban area, but in contact with the confluence of two rivers, and also a reference hospital for communities that are on the Amazonic river, so we think this “water contact” can also affects acquisition of the parasite. We have explained this better in the discussion:*

*(Lines 92-94): “Significant risk factors for stool positivity included living in a rural (unpaved) area (adjusted OR: ~~2.16~~ 1.86), whereas significant risk factors for both stool and seropositivity included living in a poor house (made of wood/leaves)”*

*(Lines 394-407): Studies evaluating specific risk factors are rare. In our cohort, adjusted risk of S. stercoralis stool positivity was higher among PLWH living in a house made of wood/leaves (considering this an indicator of low economic power), ~~or~~ in a rural area (considering here all the unpaved streets, regardless of the type of material of the house), or with a low level of education, which tallies with well-known risk factors such as poor sanitary conditions, frequent contact with fecal contaminated soil due to lifestyle habits, ~~and~~ poor healthcare access and, encompassing this, with a socioeconomic vulnerability [5,23,43,44]. Additionally, we found that study participants presenting to the Loreto Regional Hospital, an urban area in the confluence of the two main rivers, had a higher risk of S. stercoralis infection than those presenting to Iquitos Hospital. Loreto Regional Hospital is the referral hospital for people from river communities with lack of access to potable water, which may explain our results.~~The high S. stercoralis exposure rate is unsurprising given that Loreto Regional Hospital is a tertiary referral center for the enormous Amazonian governmental region of Loreto, therefore providing healthcare to people from remote areas and river communities that lack access to potable water and modern sanitation.~~*

**• 368-371: “ treatment in the previous 6 months was a protective factor for S. stercoralis stool positivity in the bivariable analysis but not in the multivariate analysis [32], probably because ivermectin (the treatment of choice for strongyloidiasis) use was uncommonly reported by our study participants…” I do not understand what this means exactly. Maybe you can rephrase it. Also, Albendazole can be effective for strongyloidiasis, if administered twice in 3 days. Maybe you will have this information ¿?**

*Answer: Thank you very much for your question. We are afraid of having some memory bias in this variable regarding to the dose of Albendazole, the main drug used in Peru for deworming, so we want to express it with caution. We have rephrased the phrase that has led to confusion with your suggestions:*

*(Lines 433-437): “…analysis [32]. While the preventive use of Albendazole (administered twice during 3 days) could explain a protective effect over S. stercoralis, many people did not remember the dosage, so this can affect the results. ~~probably because ivermectin (the treatment of choice for strongyloidiasis) use was uncommonly reported by our study participants compared with albendazole.~~*

**• Line 345: “…have hypothesized … ” Hypothesized??**

*Answer: We have rephrased the paragraph:*

*(Lines 390-393): Previous investigations in PLWH ~~Some investigators~~ [16,24,31,41] ~~have hypothesized that~~ suggest that the socioeconomic status, AIDS stage, ~~comorbid~~ alcoholism or male gender may contribute to risk for S. stercoralis infection in this group ~~in PLWH…~~”.*

**• Lines 374-375: “This dual methodology enhances diagnostic accuracy by addressing both active infections and past or subclinical exposure” I think this sentence is not correct. First, it is well known that the strongyloidiasis diagnosis benefits from a combination of techniques. Second, you cannot differentiate between active infections and past or subclinical exposure in a population living conditions you have describe as poor, related to hygiene and sanitation, where re-infection will be common.**

*Answer: We apologize for not expressing well what we wanted to say. As you comment, we wanted to highlight the fact that combination of techniques are the best way to approach S. stercoralis diagnosis. We have made the appropriate corrections.*

*(Lines 438-441): A key strength of this study is its comprehensive approach to evaluate S. stercoralis infection in PLWH, utilizing two complementary classes of tests: parasitological analysis of stool samples and serological testing. ~~This dual methodology enhances diagnostic accuracy by addressing both active infections and past or subclinical exposure.~~*

**Lines 383-385: “….as PCR has a higher sensitivity than either of the parasitological methods we employed and could identify low-level infections” This is not correct; even though the PCR is slightly more sensitive that Baermann technique in most of the studies, the test is negative in a high proportion of Baermann positives (see. Epidemiology of intestinal helminthiases in a rural community of Ethiopia: Is it time to expand control programs to include Strongyloides stercoralis and the entire community? PLoS neglected tropical diseases, 14(6), e0008315.** [**https://doi.org/10.1371/journal.pntd.0008315**](https://doi.org/10.1371/journal.pntd.0008315)**)**

*Answer: We consider this clarification to be very accurate, so we have modified the text to be more precise.*

*(Lines 450-451): “…as PCR has a high~~er~~ sensitivity ~~than either of the parasitological methods we employed~~ and, in combination with the other techniques, could help to identify low-level infections…”*

**Reviewer 2**

1. **Methods:** Not comments about these field were done by Reviewer 2.
2. **Results:** **The analysis matches the plan except where no results are given for the direction examination method which was used. The authors should mention this even if no parasites were found using the method. 3. Conclusions: Again, this could be strengthened if data were available on other helminth infections which could be incorporated in control measures.**

*Answer: Thank you for this two comments. Only 1 patient was positive for S. stercoralis in direct examination. We have added this in Results. Likewise, a section on other helminths found in feces has been added to the article.*

*(Lines 265-266): “Among the 339 participants with available stool samples, only 1 was positive in the direct exam, while 82 tested positive by the modified Baermann…”*

*(Line 274-280):*

*3.3. Other Helminths isolated in stool examination*

*Among the 339 patients with stool specimen, 17 (5.0%) were positive for hookworms, 10 (2.9%) for Ascaris lumbricoides, 2 (0.6%) for Hymenolepis nana and 1 (0.3%) for Trichuris trichiura.*

*Among the 82 patients who were positive for S. stercoralis infection in stool, 15 (18.2%) were also co-infected by hookworms, 6 (7.3%) were coinfected by Ascaris lumbricoides, and 1 (1%) was co-infected by Trichuris trichiura.*

3. **Editorial data:**

**Abstract**: **1. Change “prevalent” to important. 2. State “sociodemographic” factors instead of just factors. 3. Change “via” to “using”**

*Answer:* *Thank you for your suggestions, all have been accepted and changed in the text:*

*(Line 77): “Strongyloidiasis is an important ~~prevalent~~ but…”*

*(Line 80): “ …sociodemographic factors of Strongyloides stercoralis..”*

*(Line 84): “ …patient sera using ~~via~~ Strongyloides IgG ELISA..”*

**Line 112. Strongyloidiasis is also caused by Strongyloides fuelleborni. Authors should state that it’s caused by S. stercoralis in the study region.**

*Answer: We have specified in the text that the causative species is S. stercoralis.*

*(Lines 124-126): “Strongyloidiasis is a soil-transmitted helminthiasis caused by the parasitic nematode Strongyloides stercoralis (distributed worldwide) or Strongyloides fuelleborni (only present in some regions of Africa and Oceania)..”*

**Line 139. Delete “clinically”**

*Answer:*

*(Line 161): procedure is not ~~clinically~~ available in most patient care settings*

**Line 142. Change “tests” to “test”**

*Answer:*

*(Line 161): “serologic test~~s~~ are”*

**Line 164. Authors should state why a single stool sample was taken instead of two or three which would improve sensitivity of parasitological diagnosis**

*Answer: Due to the difficulty of obtaining even a single stool sample per patient in the complex context of Iquitos, since stool were collected at each patient's home (some of them lived far from the city or in human settlements without an address) and many patients did not notify for collection, lost the containers, moved house, were afraid of the stigma... only one stool sample was required, although the ideal would have been two or more samples. We have added this comment to the text.*

*(Line 187-189): “After providing informed consent, study participants provided a stool specimen for S. stercoralis larvae copro-parasitological examination (given the complexity of collecting it at home, only one sample per patient was required), and a blood specimen…”*

**Line 166. Change via to using**

*Answer:*

*(Line 190): “…factor variables ~~via~~ using an oral…”*

**Line 178-179. Would centrifugation of the sediment improve detection rate?**

*Answer: Thank you for this question. Modified Baermann technique was done following the quality standards of the Peruvian National Institute of Health, and did not include centrifugation of the sediment to improve detection rate, but, in some laboratories, to remove the supernatant. Therefore, the standardized technique was followed for this purpose.*

**Line 181. Change “is” to “was”**

*Answer:*

*(Line 205): “…charcoal. The mixture ~~is~~ was placed in a …”*

**Line 182-184. How was charcoal culture examined?? Stereomicroscope? Compound microscope??**

*Answer: Charcoal culture was examined with a Compound microscope. We have added this information.*

*(Line 206-207): “…The culture was examined with a compound microscope for S. stercoralis adult larvae…”*

**Line 187. Change RPM to relative centrifugal force**

*Answer: We have calculated the relative centrifugal force with 3500 rpm and a radius of 15 cm, with a result of 2058 RCF, and changed it in the text:*

*(Line 211) : Blood samples were centrifuged at ~~3500 rpm~~ 2058 RCF for 10 minutes to separate plasma.*

**Line 201. Change “presents” to “presented”**

*Answer: “…variables were presented ~~presents~~ as...”*

**Line 206. Strike “parasitological”**

*Answer: “…stercoralis ~~parasitological~~ infection were..”*

**Line 216. What about the result**

*Answer: If we have not misunderstood your point, and you are referring to results about sensitivity, specificity, positive predictive value and negative predictive value, they are represented in Table 5.*

**Line 290. “Though” should be “although”**

**Line 293. “Though” should be “although”**

*Answer:*

*(Lines 332-335): population [18–20], although ~~though~~ PLWH do not seem to have a higher risk of disseminated strongyloidiasis, probably due to their protective Th2 cytokine patterns [21–23]. S. stercoralis prevalence is likely highest in impoverished tropical areas such as the Peruvian Amazon basin, although ~~though~~ no prior*
 **Line 358-371. The authors should focus their discussion on the multivariate analysis. Also point of the study participants had very low viral loads.**

*Answer: This paragraph has been modified to focus on the multivariate analysis. We highlighted that the viral loads data were low.*

*(Lines 417-430): “seropositivity [33], while a viral load >2000 copies/ml was significatively associated with seropositivity in the bivariate, but not in the multivariate analysis. As a mere hypothesis, detectable viral load could be related to a recent diagnosis or lack of adherence to the antiretroviral treatment, what, confusingly, can lead to a greater intestinal parasite infection. As a big number of data were lost in CD4 and viral load variables, a higher sample need to be analyzed to draw stronger conclusions about the relation between an impaired immunovirological control in HIV and acquisition of the infection. ~~This could be because our patients attended their routine check-ups, and therefore had better control of the infection. Prior studies have also been inconclusive regarding the interaction between HIV and S. stercoralis infections, keeping in mind that HIV does not specifically impair Th2 immunity, in contrast to the known detrimental impacts of HTLV-1 infection and corticosteroid treatment [17]. However, in our study~~, Other previously described risk factors, including a non-heterosexual sexual orientation [25,46], ~~viral load > 2000,~~ low educational level, agricultural occupation [31], and male sex [18,43], were borderline-associated with S. stercoralis seropositivity in the bivariate analysis but not found to be significant risk…”*

4. **Summary and General Comments**

**The paper can be improved by paying attention to the following:**

**Introduction
The Introduction has not made the case for studying the epidemiology of S. stercoralis in PLWH as there is no deleterious outcome of either the virus or parasite from co-infection. However, the study is important as S. stercoralis which is treatable, can cause longstanding infections and can have complicated and fatal outcome. Diagnosing and treating S. stercoralis in PLWH remove a possible cause of severe morbidity and mortality from a vulnerable population.**

*Answer: Thank you for this point of view. As we comment in the text, PLWH could be more exposed to corticosteroids, a very known factor for hyperinfection syndrome. As you rightly point out, it is a population more vulnerable to diarrheic events, that can be chronic or require a longer recovery period. We have changed the introduction of the article to emphasize more the need of the study.*

*(Lines 145-148): Despite this, we do know that this is a vulnerable population with a higher risk for high-dose corticosteroid use (a known risk factor for hyperinfection [6]), and in which treating the parasite would reduce morbimortality.*

**Methods
These are well laid out. However, the authors mentioned that they used direct stool examination after staining with Lugol’s iodine but show no results from this method. If no parasites were detected the authors should say this as this is a Result.**

*Answer: As commented above, we have added this information to the text.*

*(Lines 265-266): “Among the 339 participants with available stool samples, only 1 was positive in the direct exam, while 82 tested positive by the modified Baermann…”*

**Results
The authors did not mention finding any other helminths using the methods at hand. This is important while discussing the matter of cross-reactivity of the ELISA. If none were found this should be stated.**

*(Line 274-280):*

*3.3. Other Helminths isolated in stool examination*

*Among the 339 patients with stool specimen, 17 (5.0%) were positive for hookworms, 10 (2.9%) for Ascaris lumbricoides, 2 (0.6%) for Hymenolepis nana and 1 (0.3%) for Trichuris trichiura.*

*Among the 82 patients who were positive for S. stercoralis infection in stool, 15 (18.2%) were also co-infected by hookworms, 6 (7.3%) were coinfected by Ascaris lumbricoides, and 1 (1%) was co-infected by Trichuris trichiura.*

**Discussion
First paragraph should be used to discuss the major finding of the study.**

*Answer: We have changed the first paragraph to the Introduction, as the second one discusses the major finding of the study.*

*(Lines 325-328):*

***Discussion***

*~~“This study provides an in-depth epidemiologic analysis of S. stercoralis in PLWH in Iquitos, Peru, and compares S. stercoralis parasitological and serological tests.~~ We detected a high prevalence of S. stercoralis infections in this population; approximately 1 in 4 PLWH had S. stercoralis larvae identified in stool and the seroprevalence was higher than 40%...”*

**The presence or absence of other helminths should be stated in the study. If none were found then other references should be cited on this to both account for cross reactivity and the approach to control of soil transmitted helminths in the area including S. stercoralis.**

*Answer: As you conveniently mentioned in this paragraph, we have also registered other intestinal parasites present in the stool sample, even if we had not included the results here so as not to prolong the text. But, as it is an interesting data, we have already added our results and discussed them. In both, the bivariate and the multivariate analysis, a significative relationship between hookworms and Strongyloides stercoralis in both, stool infection and seropositivity has been found. Previous authors have discuss this in depth, explaining that the distribution of both species could frequently be overlapped, given their biological and epidemiological similarity, as both infections are acquired by contact of the larvae with the skin when walking barefoot or bathing in rivers (Krolewiecki A, Nutman TB. Strongyloidiasis. Infect Dis Clin North Am. marzo de 2019;33(1):135-51. Fleitas PE, Travacio M, Martí-Soler H, Socías ME, Lopez WR, Krolewiecki AJ ; The Strongyloides stercoralis-hookworms association as a path to the estimation of the global burden of strongyloidiasis: A systematic review. Cappello M, editor. PLoS Negl Trop Dis. 13 de abril de 2020;14(4):e0008184; Bern C, Chavez CB, Leung F, Sanchez GM, Oré CV, Gilman RH, et al. SEROEPIDEMIOLOGY OF STRONGYLOIDIASIS IN THE PERUVIAN AMAZON. Am J Trop Med Hyg. 1 de enero de 2006;74(1):97-102). Some of this studies have therefore proposed estimating S. stercoralis from the hookworm load at the study site, in order to overcome the obstacle of the diagnostic complexity of strongyloidiasis. A cross reaction with serology because of hooworms has been feared, but when we analyzed the sensibility of the test, it remained as high as 90%, with a negative predictive value of 95%, as only one hookworm infection was found to have a positive serology in absence of S. stercoralis stool infection. In fact, as co-infection with both microorganisms is frequent, S. stercoralis could be harder to find, and treatment with Ivermectine has no major side effects, some authors suggest to treat patients with hookworms also with Ivermectine. We have included all this explanation in the abstract, the results and the discussion:*

*(Line 92-97): “Significant risk factors for stool positivity included living in a rural (unpaved) area (adjusted OR: ~~2.16~~ 1.86), whereas significant risk factors for both stool and seropositivity included living in a poor house (made of wood/leaves) (adjusted ORs: ~~2.10~~ 2.18 and 2.48, respectively), ~~or~~ in the Loreto Regional Hospital catchment area (adjusted ORs: 5.66 and 5.37, respectively), or being infected by hookworms in stool (adjusted ORs: 23.88 and 9.78, respectively). Having a low level of studies was associated with seropositivity (adjusted OR 2.42)”.*

*(Line 98-100): “Conclusion: The prevalence of S. stercoralis is high among PLWH in Iquitos, especially among those living in conditions of socioeconomic vulnerability ~~rural areas~~ or co-infected with hookworms.”*

*(Line 242-243): “Same analysis was realized also including other helminths infections, in other to rule out cross-reactions”.*

*(Line 274-280):*

*3.3. Other Helminths isolated in stool examination*

*Among the 339 patients with stool specimen, 17 (5.0%) were positive for hookworms, 10 (2.9%) for Ascaris lumbricoides, 2 (0.6%) for Hymenolepis nana and 1 (0.3%) for Trichuris trichiura.*

*Among the 82 patients who were positive for S. stercoralis infection in stool, 15 (18.2%) were also co-infected by hookworms, 6 (7.3%) were coinfected by Ascaris lumbricoides, and 1 (1%) was co-infected by Trichuris trichiura.*

*(Lines 297-303): “…After adjusting by sex, age, and variables with p values ≤ 0.05 in the bivariate analysis (****Table 4****), ~~two~~ four variables had a significant association with S. stercoralis seropositivity: belonging to Loreto Regional Hospital catchment area (adjusted OR~~: 5.37~~ 3.88), ~~and~~ living in a house made of wood/leaves (adjusted OR: ~~2.48~~ 2.82), having hookworms in stools (adjusted OR: 9.78) ~~(~~****~~Figure 3b~~****~~),~~ and having a low level of education (illiteracy or primary school) (adjusted OR 2.42) (****Figure 3b****). The model’s Cox–Snell R^2^ value was ~~0.08~~ 0.17 and the Nagelkerle R^2^ value was ~~0.12~~ 0.23, with an AUC of ~~0.69~~ 0.72 (95% CI ~~0.62–0.76~~ 0.67 – 0.78, p < 0.001).*

*(Lines 314-317): Only one hookworm infection was found to have a positive serology in absence of S. stercoralis stool infection. A cross reaction with serology because of hookworms has been feared, but when we analyzed the sensitivity of the test including this variable, it remained as high as 90% (IC:0.81-0.95), with a negative predictive value of 95% (0.91-0.98).*

*(Lines 408-415): “Hookworm infection was significantly related to infection in stool or seropositivity by S. stercoralis. This have been discussed in previous studies, as the distribution of both species could frequently be overlapped, given their biological and epidemiological similarity, being suggested as a reliable path to the estimate the global burden of strongyloidiasis [10,44].” Although there was an understandable fear of cross-reactions in serology due to other helminths, only one case showed a positive serology with stool positivity for hookworm rather than S. stercoralis, with no impact on the ELISA sensitivity/negative predictive values.*

*** Note to the reviewer: When the multivariate analysis was repeated with the new parameters, a significant value was obtained for the variable "low educational level," which had not been found significant in the first analysis. This is a logical risk factor for an infection associated with low socioeconomic status, so we have added this finding to the text:

*(Lines 91-100):* “…*Significant risk factors for stool positivity included living in a rural (unpaved) area (adjusted OR: ~~2.16~~ 1.86), whereas significant risk factors for both stool and seropositivity included living in a poor house (made of wood/leaves) (adjusted ORs: ~~2.10~~ 2.18 and 2.48, respectively), ~~or~~ in the Loreto Regional Hospital catchment area (adjusted ORs: 5.66 and 5.37, respectively), or being infected by hookworms in stool (adjusted ORs: 23.88 and 9.78, respectively). Having a low level of studies was associated with seropositivity (adjusted OR 2.42).*

*Conclusion: The prevalence of S. stercoralis is high among PLWH in Iquitos, especially among those living in conditions of socioeconomic vulnerability ~~rural areas~~ or co-infected with hookworms.”*

*(Lines 286-293): “After adjusting for sex, age, and variables with p values ≤ 0.05 in the bivariate analysis* ***(Table 3),*** *the characteristics most strongly associated with parasitological infection of S. stercoralis in PLWH were belonging to the Loreto Regional Hospital catchment area (adjusted OR: ~~5.66~~ 5.43), living in a rural area (adjusted OR: ~~2.16~~ 1.86), ~~and~~ living in a house made of wood/leaves (adjusted OR: ~~2.10~~ 2.18) and having hookworms in stools (adjusted OR: 23.88) (****Figure 3a****). The model’s Cox–Snell R^2^ value was ~~0.11~~ 0.18 and the Nagelkerle R^2^ value was ~~0.17~~ 0.27, with an AUC of ~~0.73~~ 0.76 (95% CI ~~0.67–0.79~~ 0.70 – 0.82, p < 0.001).”*

*(Lines 297-303): “ After adjusting by sex, age, and variables with p values ≤ 0.05 in the bivariate analysis (****Table 4****), ~~two~~ four variables had a significant association with S. stercoralis seropositivity: belonging to Loreto Regional Hospital catchment area (adjusted OR~~: 5.37~~ 3.88), ~~and~~ living in a house made of wood/leaves (adjusted OR: ~~2.48~~ 2.82), having hookworms in stools (adjusted OR: 9.78) ~~(~~****~~Figure 3b~~****~~),~~ and having a low level of education (illiteracy or primary school) (adjusted OR 2.42) (****Figure 3b****). The model’s Cox–Snell R^2^ value was ~~0.08~~ 0.17 and the Nagelkerle R^2^ value was ~~0.12~~ 0.23, with an AUC of ~~0.69~~ 0.72 (95% CI ~~0.62–0.76~~ 0.67 – 0.78, p < 0.001).”*

*(Lines 395-400): “…stool positivity was higher among PLWH living in a house made of wood/leaves (considering this an indicator of low economic power), or in a rural area (considering here all the unpaved streets, regardless of the type of material of the house), or with a low level of education, which tallies with well-known risk factors such as poor sanitary conditions, frequent contact with fecal contaminated soil due to lifestyle habits, and poor healthcare access and, encompassing this, with a socioeconomic vulnerability.”*

*The Tables 3, 4 and the Figure 3a - 3b have been changed adding this data:*

**Table 3**

| ART adherence <= 95% (bad adherence), % (n/N) | 12.2 (10/82) | 11.7 (30/257) | 1.09 (0.50-2.36) | 0.832 |
| --- | --- | --- | --- | --- |
| Hookworms in stool | 18.3 (15/82) | 0.8 (2/257) | 28.55 (6.37-127.90) | **<0.001** |

**Table 4**

| Adherence <= 95% (bad adherence), % (n/N) | 12.8 (29/227) | 11.1 (34/307) | 1.18 (0.69-2.02) | 0.547 |
| --- | --- | --- | --- | --- |
| Hookworms in stool | 7.0 (16/227) | 0.7 (2/307) | 10.47 (2.37-46.24) | **<0.001** |
