# Supplementary material for: High prevalence of Strongyloides stercoralis in people living with HIV: A critical health challenge in the Peruvian Amazon Basin
Source: PLoS Negl Trop Dis. 2025 Jul 15;19(7):e0013231. doi: 10.1371/journal.pntd.0013231 (PMC12262856; doi:10.1371/journal.pntd.0013231)
Supplement: Attachment — Submitted filename: Response_to_reviewers_auresp_2.docx [file pntd.0013231.s005.docx]

Esteemed editor,

We sincerely appreciate the time and dedication you and the reviewers have invested in evaluating our manuscript. We have carefully revised the article and incorporated the latest suggestions, that have significantly improved the quality of the article. The modifications are highlighted in red within the text. Below, we provide detailed responses and clarifications for each of the points raised:

**Please, revise accurately the newly added paragraphs - some sentences are not clear and need grammar editing**.

*Answer: Thank you very much for your valuable feedback and for highlighting the need to improve the clarity and grammar of the manuscript. We agree that some paragraphs could be grammatically improved, so we appreciate your attention to this important aspect. In response, we have carefully revised and edited the new paragraphs in the Discussion section to improve readability and ensure greater grammatical accuracy. The revised version is included below for your review.*

*Should you still find any paragraph unclear or imprecise, we would be grateful if you could kindly indicate which one(s), so that we may focus our efforts accordingly and address them more specifically.*

*(Lines 359-372): “ In areas of high endemicity, Strongyloides crude antigen IgG ELISA is known to have a lower specificity compared to reference stool tests because (1) Strongyloides IgG antibodies persist for many years after treatment and (2) the crude antigen lysate allows for cross-reactivity with other helminths that can be co-endemics with S. stercoralis [40]. Besides, ~~On the other hand~~, ELISA´s sensitivity ~~of ELISA~~ ~~could be affected~~ may be reduced in patients with AIDS due to impaired immune responses to the parasite. ~~AIDS patients, who may have a diminished immune response to the parasite~~ ~~[41], even if this population in our cohort was low (13.9%). However,~~ ~~i~~. In our study, the specificity of the ELISA was close to 70%, similar to a study of pregnant women in Iquitos performed with the same test [10],* *probably due to the persistence of antibodies in an endemic area (only one patient had a positive ELISA for S. stercoralis with a discordant parasite in stool). About ~~this~~ ELISA’s sensitivity ~~specificity~~ and negative predictive value, they were greater than 90%, ~~though in a~~ ~~similar~~ higher than in the previously cited study, where it ~~of pregnant women from Iquitos utilizing the same ELISA, specificity~~ only reached 70% [10]. The higher cutoff point that we chose for this study could be responsible for this improvement.”*

*(Lines 381-407): “Studies evaluating specific risk factors are rare. In our cohort, adjusted risk of S. stercoralis stool positivity was higher among PLWH living in a house made of wood/leaves (used as a proxy for low socioeconomic status ~~considering this an indicator of low economic power~~), in a rural area (defined by the presence of unpaved streets, regardless of housing material) ~~considering here all the unpaved streets, regardless of the type of material of the house~~), or with a low level of education. These findings are consistent with well-established risk factors, including poor sanitation, contact with fecal contaminated soil due to lifestyle practices, limited access to healthcare, and overall socioeconomic vulnerability ~~which tallies with well-known risk factors such as poor sanitary conditions, frequent contact with fecal contaminated soil due to lifestyle habits, poor healthcare access and, encompassing this, with a socioeconomic vulnerability~~ [5,23,43,44]. Additionally, we found that study participants attending ~~presenting to the~~ Loreto Regional Hospital —an urban area in the confluence of ~~the~~ two major ~~main~~ rivers—, had a higher risk of S. stercoralis infection than those attending ~~presenting to the~~ Iquitos Hospital. Loreto Regional Hospital is the referral hospital for people from river communities with limited ~~lack of~~ access to potable water, which may explain our results. This data also could be influenced by the higher amount of patients collected in this Hospital. Hookworm infection in stool was significantly ~~related~~ associated with both ~~to~~ S. stercoralis infection and seropositivity. ~~This have been discussed in previous studies, as the distribution of both species could frequently be overlapped, given their biological and epidemiological similarity, being suggested as a reliable path to the estimate the global burden of strongyloidiasis~~ This co-occurrence has been discussed in previous literature, as the distribution of both helminths often overlaps due to similar biological and epidemiological characteristics. Consequently, hookworm has been proposed as a proxy indicator for estimating the global burden of strongyloidiasis [11,45]. Although cross-reactivity in serology remains a concern, it did not affect ELISA´s specificity in our cohort, as previously discussed. ~~there was an understandable fear of cross-reactions in serology due to other helminths, only one case showed a positive serology with stool positivity for hookworm rather than S. stercoralis, with no impact on the ELISA sensitivity/negative predictive values.”~~*

*(Lines 408- 428): “In our study, AIDS stage was not significantly associated with either S. stercoralis stool positivity or seropositivity [33]. A ~~while a~~ viral load >2000 copies/ml was ~~significatively~~ significantly associated with seropositivity in the bivariate analysis, ~~but~~ though not in the multivariate analysis. However, the substantial amount of missing data on CD4 count and viral load limits the strength of conclusions regarding the association between poor immunovirological control and infection risk.~~. As a mere hypothesis, detectable viral load could be related to a recent diagnosis or lack of adherence to the antiretroviral treatment, what, confusingly, can lead to a greater intestinal parasite infection. As a big number of data were lost in CD4 and viral load variables, a higher sample need to be analyzed to draw stronger conclusions about the relation between an impaired immunovirological control in HIV and acquisition of the infection~~. Other previously described risk factors, including a non-heterosexual sexual orientation [25,46], low educational level, agricultural occupation [31], and male sex [18,43], were borderline-associated with S. stercoralis seropositivity in the bivariate analysis but not found to be significant risk factors in the multivariate analysis. ~~We also found that receipt of deworming treatment in the previous 6 months~~ Deworming treatment within the past six months ~~was a~~ appeared protective ~~factor for~~ against S. stercoralis stool positivity in the bivariable analysis but ~~not~~ was not significant in the multivariate analysis [33]. ~~While the preventive use of Albendazole (administered twice during 3 days) could explain a protective effect over S. stercoralis, many people did not remember the dosage, so this can affect the results~~. Although albendazole (administered twice over three days) may explain this effect, many participants could not recall the exact drug dosage, which may have influenced the results.”*

*(Lines 435-454): “This study has several limitations. First, we collected and analyzed only a single stool sample, which may have underestimated the true prevalence of S. stercoralis [47]. ~~The use of~~ Analyzing three ~~stool~~ samples could have improved the detection rate of active infection [48]. ~~Countering~~ To mitigate this limitation, we used two different parasitological techniques to enhance the sensitivity of larval detection. Second, ~~not using~~ the absence of molecular techniques, (e.g., PCR), which are highly sensitive and useful for identifying low-level infections, may have limited diagnostic accuracy. ~~likely limits diagnostic accuracy, as PCR has a high sensitivity and, in combination with the other techniques, could help to identify low-level infections. Another limitation would be that our study mainly captured only~~ Additionally, our cohort primarily included PLWH who ~~attended their routine follow-up~~ were actively engaged in routine care~~, leaving out PLWH who did not attend for care, in whom the prevalence of Strongyloides could be higher.~~ , possibly underrepresenting individuals not accessing care—who may be at greater risk of infection. Finally, the large amount of missing data on CD4 and viral load hindered our ability to fully explore associations between immunovirological control and infection risk. While our findings highlight a high burden of S. stercoralis in the Amazon, they may not be generalizable to regions with different epidemiological contexts. ~~Furthermore, we lost a significant data about CD4 and viral load, important to draw conclusions about risk factors in PLWH with poor immunovirological control. Finally, while our results emphasize the high burden of S. stercoralis infection in the Amazon, this high prevalence may not be generalizable to other geographic areas with distinct epidemiological characteristics.~~*

**Introduction (lines 135-138) - I suggest revising this paragraph as follows: "The World Health Organization (WHO) includes Strongyloides among the soil transmitted helminthiases targeted for improved control by 2030; for this purpose, specific guidelines have been published recently. However, the WHO [...]"**

*Answer: Thank you very much for your kind suggestion. We believe the paragraph modification you proposed is really appropriated, so we have re-written it following your indications:*

*(Lines 133-136): The World Health Organization (WHO) includes Strongyloides among the soil transmitted helminthiases targeted for improved control by 2030; ~~. Specifically,~~ for this purpose, specific guidelines have been published recently. However, the organization denounces the need for more evidence to optimize public health programs for strongyloidiasis [6].*

**Methods, paragraph 2.3: I would delete the first sentence ("We established...")**

*Answer: Thank you for the suggestion. We have deleted the first sentence:*

*“(Lines 189-190): ~~We established stool examination as the reference standard diagnostic method for S. stercoralis.~~ Each fecal specimen was analyzed using three techniques…”*

**Please, when refering to larvae avoid the term "adult", which generates confusion and is not appropriate; use either lavae or adult worms.**

*Answer:*

*(Line 202): “…for S. stercoralis adult worm ~~larvae~~…”*

*(Line 203): “… days to observe adult worm ~~larvae~~ before discarding…”*

**Last paragraph of Results: it is sufficient to report that only one S stercoralis seropositive individuals had no S stercoralis but hookworm larvae in stool. There is no need to re-calculate sensitivity (by the way, probably it would make more sense to recalculate specificity...) and predictive values.**

*Answer: Thank you for your insightful comment. We completely agree that specificity is the critical aspect to consider in a cross-reaction because of other parasitic infections. However, the reviewer specifically asked about its potential impact on sensitivity: “It would be important to mention that other STHs, e.g., hookworm, can be co-infecting the patients, and the relationship with positive serology in those patients, that is, the impact of this fact on the sensitivity of the test.” That said, we agree with your suggestion and will exclude the new calculation, retaining only the original analysis:*

*(Lines 305-308): “Only one hookworm infection was found to have a positive serology in absence of S. stercoralis stool infection. ~~A cross reaction with serology because of hookworms has been feared, but when we analyzed the sensitivity of the test including this variable, it remained as high as 90% (IC:0.81-0.95), with a negative predictive value of 95% (0.91-0.98).~~”*
